# 192. Tolerability Outcomes of Multi-drug Antibiotic Treatment for Pulmonary Nontuberculous Mycobacterial Disease due to *Mycobacterium avium* Complex in U.S. Medicare Beneficiaries with Bronchiectasis

**DOI:** 10.1093/ofid/ofab466.192

**Published:** 2021-12-04

**Authors:** Jennifer Ku, Emily Henkle, Kathleen F Carlson, Miguel Marino, Sarah K Brode, Theodore K Marras, Kevin L Winthrop, Kevin L Winthrop

**Affiliations:** 1 Oregon Health & Science University, Portland, OR; 2 University Health Network and University of Toronto, Toronto, Ontario, Canada

## Abstract

**Background:**

Nontuberculous mycobacteria (NTM), most frequently *Mycobacterium avium* complex (MAC), cause increasingly common pulmonary infections. Treatment interruptions and early discontinuation are common in MAC therapy, but population-based data on treatment outcomes are severely lacking. We examined tolerability outcomes of guideline-based 3-drug therapies (GBT) targeted for pulmonary MAC infection.

**Methods:**

Among beneficiaries with bronchiectasis (ICD-9-CM 494.0 or 494.1) in U.S. Medicare data (01/2006 – 12/2014), we identified first-time MAC GBT therapy users, excluding those with cystic fibrosis, HIV, or a history of organ transplant. MAC GBT was defined as an overlapping prescription of ≥ 28-day supply of a macrolide, ethambutol and rifamycin. Using Cox regression methods, we compared time-to-regimen change or discontinuation within 12 months of therapy start in the following groups: 1) azithromycin-ethambutol-rifamycin vs. clarithromycin-ethambutol-rifamycin; 2) macrolide-ethambutol-rifampin vs. macrolide-ethambutol-rifabutin; and 3) azithromycin-ethambutol-rifampin vs. clarithromycin-ethambutol-rifabutin.

**Results:**

We identified 4,626 GBT therapy users (mean 77.9 years [s.d. 6.1], female [77.7%], and non-Hispanic white [87.2%]). Overall, the rate of regimen change or discontinuation was higher in the clarithromycin-based regimens compared to azithromycin-containing regimens, and in rifabutin-containing regimens compared to rifampin-containing regimens. The rate of drug regimen change or discontinuation was 65% greater in the clarithromycin-ethambutol-rifabutin group compared to the azithromycin-ethambutol-rifampin group (adjusted hazard ratio: 1.64, 95% CI: 1.43, 1.64) (**Table 1**, **Figure 1**).

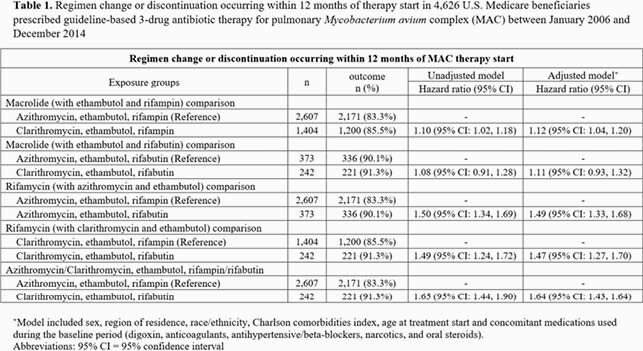

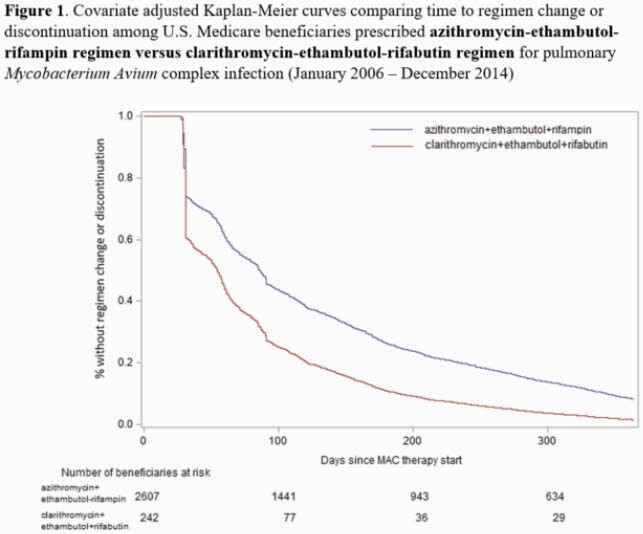

**Conclusion:**

Azithromycin-based regimens and rifampin-containing regimens were less likely to be changed or discontinued within 12 months of therapy compared to clarithromycin-based regimens and rifabutin-containing regimens, respectively. More research is needed to identify factors associated with early treatment change or discontinuation.

**Disclosures:**

**Emily Henkle, PhD, MPH**, **AN2** (Consultant, Advisor or Review Panel member)**Zambon** (Advisor or Review Panel member) **Theodore K. Marras, MD**, **Astra Zeneca** (Speaker’s Bureau)**Insmed** (Scientific Research Study Investigator)**Novartis** (Speaker’s Bureau)**RedHill** (Consultant)**Spero** (Consultant) **Kevin L. Winthrop, MD, MPH**, **Insmed** (Consultant, Grant/Research Support)**Paratek** (Consultant)**RedHill** (Consultant)**Spero** (Consultant) **Kevin L. Winthrop, MD, MPH**, **Insmed** (Consultant, Research Grant or Support)**Paratek** (Consultant)**RedHill Biopharma** (Consultant)**Spero** (Consultant)

